# Butein suppresses breast cancer growth by reducing a production of intracellular reactive oxygen species

**DOI:** 10.1186/1756-9966-33-51

**Published:** 2014-06-11

**Authors:** Sung-Gook Cho, Sang-Mi Woo, Seong-Gyu Ko

**Affiliations:** 1 Department of Preventive Medicine, College of Korean Medicine, Kyung Hee University, 1 Hoegi, Seoul 130701, Korea

**Keywords:** Butein, Breast cancer, Reactive oxygen species, AKT, Apoptosis

## Abstract

**Background:**

Butein has various functions in human diseases including cancer. While anti-cancer effects of butein have been revealed, it is urgent to understand a unique role of butein against cancer. In this study, we demonstrate that butein inhibition of reactive oxygen species (ROS) production results in suppression of breast cancer growth.

**Methods:**

Different breast cancer cell lines were treated with butein and then subjected to cell viability and apoptosis assays. Butein-sensitive or -resistant breast cancer cells were injected into mammary fat pads of immunocompromised mice and then butein was injected. Breast cancer cells were categorized on the basis of butein sensitivity.

**Results:**

Butein reduced viabilities of different breast cancer cells, while not affecting those of HER2-positive (HER2^+^) HCC-1419, SKBR-3 and HCC-2218 breast cancer cells. Butein reduction of ROS levels was correlated with apoptotic cell death. Furthermore, butein reduction of ROS level led to inhibitions of AKT phosphorylation. N-acetyl-L-cysteine (NAC), a free radical scavenger, also reduced ROS production and AKT phosphorylation, resulting in apoptotic cell death. In contrast, inhibitory effects of both butein and NAC on ROS production and AKT phosphorylation were not detected in butein-resistant HER2^+^ HCC-1419, SKBR-3 and HCC-2218 cells. In the *in vivo* tumor growth assays, butein inhibited tumor growth of butein-sensitive HER2^+^ BT-474 cells, while not affecting that of butein-resistant HER2^+^ HCC-1419 cells. Moreover, butein inhibition of ROS production and AKT phosphorylation was confirmed by *in vivo* tumor growth assays.

**Conclusions:**

Our study first reveals that butein causes breast cancer cell death by the reduction of ROS production. Therefore, our finding provides better knowledge for butein effect on breast cancer and also suggests its treatment option.

## Background

Butein (2′,3,4,4′- 2′,4′,3,4- or 3,4,2′,4′-tetrahydroxychalcone) can be isolated in various plants including *Toxicodendron vernicifluum* (*Rhus verniciflua*), *Semecarpus anacardium* and *Dalbergia odorifera*, and has different biological functions [1-8]. Butein has been known to inhibit cancer development or progression, while information on its anti-cancer effect is limited in particular cell lines or experimental conditions. Butein inhibited skin carcinogenesis in female CD-1 mice by inhibiting epidermal 12-lipoxygenage activity, while not affecting PKC activation [2]. Butein also induced apoptotic cell death in colon adenocarcinoma cells [9], leukemic cells [6,10], lymphoma cells [11], multiple myeloma cells [12], breast cancer cells [13,14], osteosarcoma cells [15], hepatocellular carcinoma cells [16,17], prostate cancer cells [18,19], uveal melanoma cells [20], neuroblastoma cells [21], and malignant pleural mesothelioma cells [22]. In addition, butein inhibited cell migration and invasion in hepatocellular carcinoma cells [23], bladder cancer cells [24] and pancreatic and breast cancer cells [8]. Those studies indicate that butein may suppress both primary tumor growth and distant metastases, while most of those studies were conducted *in vitro*. Meanwhile, butein inhibited glycochenodeoxycholic acid-induced apoptosis in hepatocytes [25] and inflammatory responses in TNFα-induced intestinal epithelial cells [26]. Therefore, butein may also protect tumorigenic process.

Breast cancer is one of most common cancers. While breast cancer is highly heterogeneous, it is broadly categorized into luminal and basal subtypes on the basis of gene expression patterns and classified into three major therapeutic subtypes: estrogen receptor-positive (ER^+^; receiving endocrine therapy), HER2-positive (HER2^+^; targeting HER2), and triple-negative (lacking expression of ER, progesterone receptor and HER2; receiving chemotherapy) [27,28]. While anti-cancer effects of butein were reported as mentioned above, its anti-cancer effect on breast cancer has not been clearly proven. Butein inhibited a growth of ER^+^ MCF-7 cells where aromatase was stably overexpressed [13]. Butein also blocked CXCL12-induced migration and invasion of HER2^+^ SKBR-3 cells by repressing NF-κB-dependent CXCR4 expression [8]. In triple-negative (triple-negative breast cancer, TNBC) MDA-MB-231 cells, butein induced apoptosis through a generation of reactive oxygen species (ROS) and deregulation of ERK1/2 and p38MAPK [14]. Nevertheless, we could not rule out that butein effects on those breast cancer cell types reflect breast cancer subtype- or cell type-restricted or experimental condition-specific responses, although cell lines used in literatures above are widely used as representatives of particular breast cancer subtypes. Thus, it is urgent to clearly understand butein effect on breast cancer.

Butein effect on ROS generation has been investigated in various physiological and pathological conditions with different cell types [14,21,29-36]. While butein has been reported to induce ROS generation in MDA-MB-231 breast cancer cells [14], its effect on ROS generation is inconsistent in different biological conditions [3,8,15,20,30,32]. Butein increased ROS level in particular cell types or experimental conditions, resulting in apoptotic cell death [14,21,31]. However, butein inhibited ROS level increased by particular environmental cues such as ethanol, H_2_O_2_ and TNF-α [29,30,32-36]. Furthermore, it has been revealed that ROS regulates breast cancer progression [37,38]. Thus, butein effect on the alteration of ROS level in breast cancer cells is still unclear.

In this study, we investigated butein effects on different types of breast cancer cells. Our data showed that butein caused apoptotic cell deaths of breast cancer cells, while not affecting luminal HER2^+^ SKBR-3, HCC-1419 and HCC-2218 cell lines. Furthermore, butein-induced cell death resulted from reductions of ROS level and AKT phosphorylation. Butein-resistant phenotype of luminal HER2^+^ SKBR-3, HCC-1419 and HCC-2218 breast cancer cell lines was due to no alteration of ROS level and AKT phosphorylation status. Consistently, butein suppressed *in vivo* breast tumor growth with the reduction of ROS level. Therefore, our present study provides better knowledge for butein role against breast cancer.

## Methods

### Reagents and cell lines

**Figure 1:**
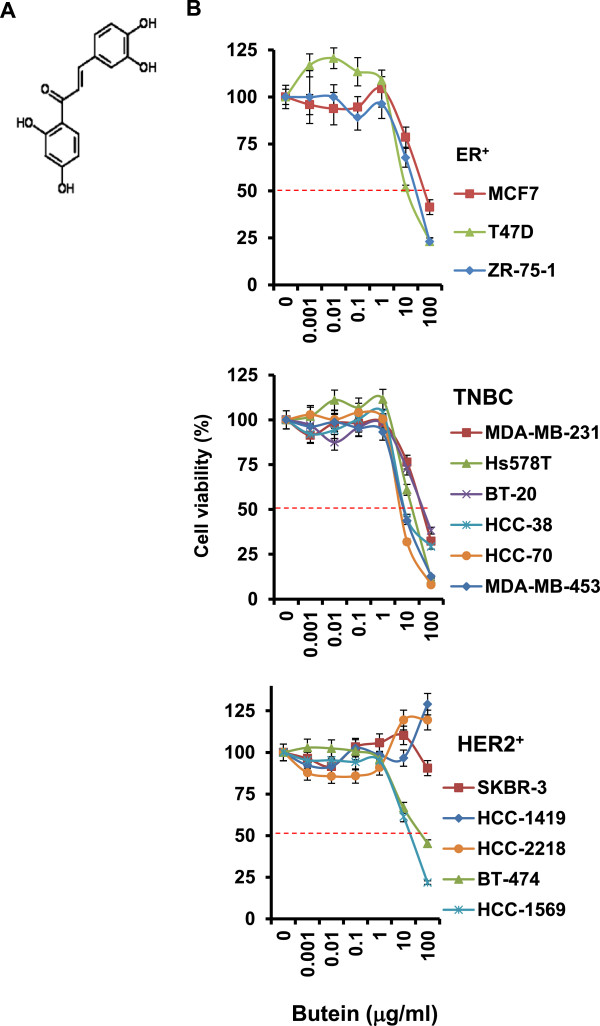
**Butein effect on viabilities of different breast cancer cell lines. (A)** Butein structure. **(B)** Butein inhibition of breast cancer cell viabilities. Breast cancer cells were treated with different doses of butein (0-100 μg/ml) for 48 hours and then subjected to MTT assays. Red bars indicate 50% of cell viability. Experiments were done in quadruplicate and repeated three times independently. Bars indicate the mean ± SEM.

Butein was obtained from Santa Cruz Biotechnology. Butein structure is presented in Figure 1A. Epidermal growth factor (EGF) was purchased from R&D systems (Minneapolis, MN, USA). N-acetyl-L-cysteine (NAC) was obtained from Sigma-Aldrich (St. Louis, MO, USA). MCF-10A, MCF-7, T47D, SKBR-3, MDA-MB-231 and BT-20 cells were obtained from American Type Culture Collection (ATCC; Manassas, VA, USA). MDA-MB-453, BT-474, BT-20, HCC-38, Hs578T, HCC-70, HCC-1395, HCC-1419, HCC-1569, HCC-2218 and ZR-75-1 cells were purchased from Seoul National University Cell Bank (Seoul, Korea). MCF-7, SKBR-3, Hs578T and MBA-MB-231 lines were cultured in DMEM supplement with 10% fetal bovine serum and 1% antibiotics. T47D, MDA-MB-453, BT-20, BT-474, HCC-38, HCC-70, HCC-1419, HCC-1569, HCC-2218 and ZR-75-1 cells were maintained in RPMI-1640 with 10% fetal bovine serum and 1% antibiotics.

### *In vitro* studies

For cell viability, cells were cultured in 96 well plates, treated with different concentrations of butein, and subjected to the MTT assays. Experiments were performed in quadruplicate and repeated three times independently. Data were presented as the mean ± SEM. P-values less than 0.05 were considered statistically significant. Cells were treated with butein for 24 hours and then subjected to the Annexin V-FITC Early Apoptosis Detection Kit (Cell Signaling, Danvers, MA, USA). Experiments were performed by manufacturer’s recommendations and repeated independently three times. Data presented as the mean ± SD. ROS levels were measured using 10 μM of H_2_DCF-DA (2’,7’-dichlorofluorescin diacetate; Molecular Probes, Eugene, OR, USA). Cells were treated with butein for 5 minutes and then treated with H_2_DCF-DA for another 1 hour. Flow cytometry experiments were performed in triplicate and repeated three times independently. For western blotting, cells were lyzed with RIPA buffer. For tissue samples, tissues were homogenized and then protein was extracted in RIPA buffer. 30 μg of protein was loaded. Antibodies for p-SRC, p-ERK1/2, p-AKT, SRC, ERK1/2, AKT, and actin were purchased from Cell Signaling (Danvers, MA, USA). Levels of phosphorylated forms of SRC, ERK and AKT were normalized with Actin levels in Image J software (NIH, rsbweb.nih.gov/ij/) and levels in untreated samples were set by 1. Data were then put in MultiExperiment Viewer (MeV) software (http://www.tm4.org/mv.html) to generate heat map to visualize and to analyze correlated clusters between cell lines [39]. In brief, non-negative matrix factorization (NMF) analysis using data for normalized phosphorylation levels of SRC, ERK and AKT in different cell lines treated with butein or not was done in MeV software. Rank range was 2 to 4, the highest cophenetic correlation value was 0.77221833, and a number of runs were 10.

### *In vivo* studies

For xenograft mouse tumor growth assays, 1x10^6^ MDA-MB-231 cells mixed with 10% matrigel (R&D Biosystems) were injected into the 4^th^ inguinal mammary fat pads of nude (*nu*/*nu*) mice (the Orient, Korea). Five mice were examined for each group. Tumor volume (V = D^2^xL/2; V: volume, D: distance, L: length) and mouse weight were measured two times a week. Butein at 10 μg/mL or PBS was subcutaneously injected two times a week. At day 20 post injection, mice were euthanized and tumors were fixed with 4% formaldehyde. Tumor tissues paraffin-embedded were sectioned at 10 μm and subjected to the immunohistochemistry (IHC). Tissues were deparaffinized and antigen was retrieved by 8 M Urea. TUNEL assays were performed using TACS® 2 TdT DAB kit (Trevigen, Gaithersburg, MD, USA). For BrdU incorporation assays, mice were intraperitoneally injected with 300 μL of BrdU solution (BD Pharmingen, San Diego, CA, USA) for 3 hours before being sacrificed. Anti-BrdU antibody (AbCam, Cambridge, UK) was used in 1: 1,000 dilution. BrdU- or TUNEL-stained cell numbers were counted in four fields randomly chosen in tumor tissues (n = 5/group). In addition, apoptotic cells in tumor cohorts were observed using anti-cleaved caspase-3 antibody. Phosphorylated AKT was detected using anti-pAKT antibody. To measure ROS levels *in vivo*, tumor tissues were embedded and frozen in OCT compound. Tissue sections at 20 μm were stained with 10 μmol/L of H_2_DCF-DA (Molecular Probes, Eugene, OR, USA) for 1 hour and then images were taken by the fluorescent microscope. Fluorescent cells were counted in four fields randomly chosen and repeated in 5 different tumor burdens. In addition, the intensity of fluorescence was measured at the same exposure level. Data measured represent the mean ± SEM. Photographs are representative images. Tumor tissues were homogenized and lyzed with RIPA buffer. To detect phosphorylated AKT in tumor tissues, western blots with anti-pAKT antibody and anti-AKT antibody were performed.

### Statistics

GraphPad Prism was used to test statistics (T-test or Duncan’s post-hoc ANOVA). P-values less than 0.05 were considered statistically significant.

## Results

### Butein causes apoptosis of breast cancer cells

Various breast cancer cell lines were categorized by characteristics described in the previous study [27,28] were treated with different doses of butein (1 ng/ml to 100 μg/ml) for 48 hours and then subjected to MTT assays. Butein reduced viabilities of almost all breast cancer cell lines tested, whereas it did not affect viabilities of luminal HER2^+^ HCC-1419, HCC-2218 and SKBR-3 breast cancer cells (Figure 1B).

**Figure 2:**
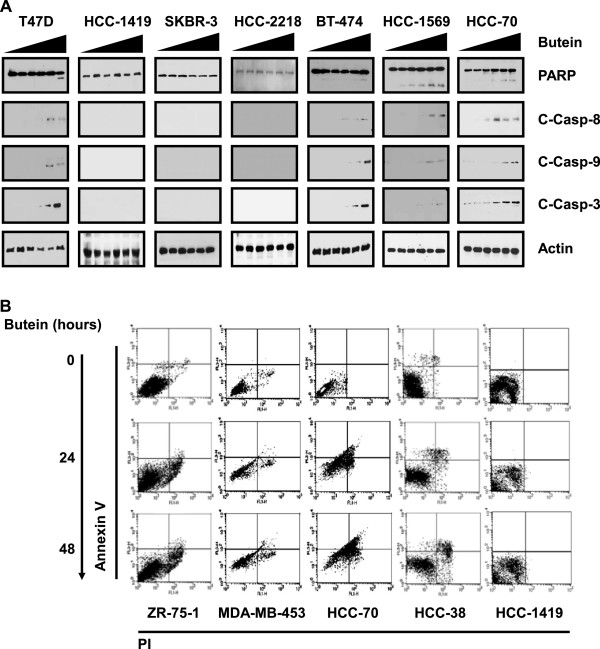
**Butein induction of apoptosis. (A)** Breast cancer cells were treated with butein at 0-100 μg/ml (left triangles) for 24 hours and then cleavages of PARP and Caspases were examined with appropriate antibodies in western blots. Actin was detected as a loading control. **(B)** Breast cancer cells were treated with butein at 10 μg/ml for 24 hours and then stained with Annexin V and PI. Cells were then counted using flow cytometry. Experiments were performed in triplicate and data present the mean ± SD.

Next, we examined butein effect on apoptosis in breast cancer cell lines. Consistent with MTT data, butein treatment for 24 hours caused cleavages of PARP and Caspases (Caspase-8, −9 and −3) in T47D (luminal, ER^+^), HCC-70 (basal A, TNBC), BT-474 (luminal, ER^+^/PR^+^/HER2^+^) and HCC-1569 (basal A, HER2^+^), while not affecting those in luminal HER2^+^ HCC-1419, HCC-2218 and SKBR-3 cells (Figure 2A). Likewise, butein at 10 μg/ml for 24 hours increased Annexin V-positive and PI-positive populations in T47D, HCC-70, BT-474 and HCC-1569 cells; whereas it did not affect HCC-1419, HCC-2218 and SKBR-3 cells (Figure 2B). Thus, our data indicate that butein causes apoptosis of breast cancer cells, while luminal HER2^+^ HCC-1419, HCC-2218 and SKBR-3 breast cancer cells are resistant to butein.

### Butein effect on apoptotic cell death of breast cancer cells is related with an alteration of ROS levels

**Figure 3:**
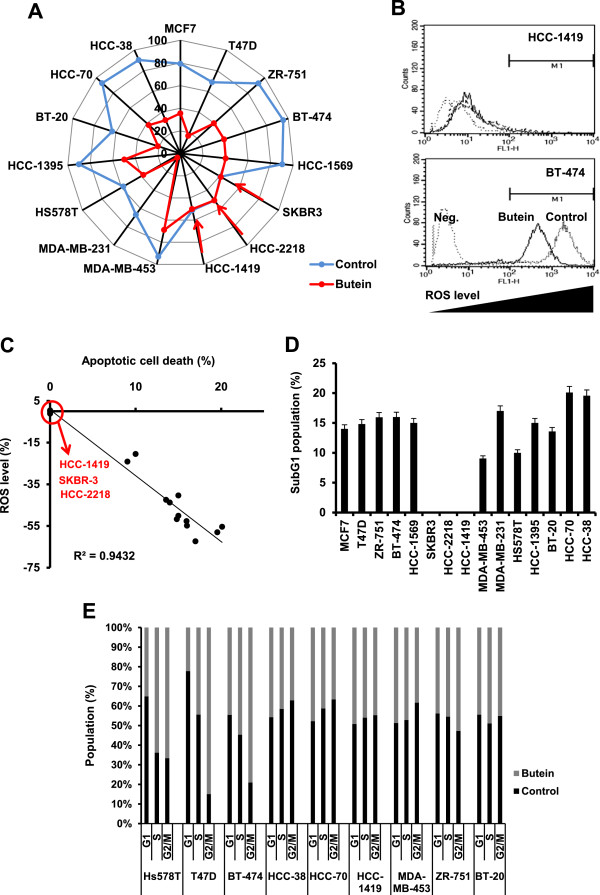
**Butein effects on ROS generation. (A)** Butein alteration of ROS level. 100% stacked bars indicate ROS levels in the cells treated with butein or not. Cells were treated with butein at 10 μg/ml for 5 minutes and incubated with H_2_DCF-DA for another 1 hour. ROS levels were measured using flow cytometry. Experiments were independently repeated three times. Red arrows indicate ROS levels in HER2^+^ breast cancer cells. **(B)** Representative data for butein-altered ROS levels in HER2^+^ breast cancer cells either sensitive (BT-474) or resistant (HCC-1419) to butein. Negative control (Neg.) indicates no treatment of ROS indicator. Left triangles indicate the increase of ROS levels. **(C)** Correlations between percentages of reduced ROS levels and percentages of apoptotic cell death. A red circle indicates butein-resistant and luminal HER2^+^ breast cancer cells. (D-E) Butein effect on cell cycle profiles. Cells were treated with 10 μg/ml of butein for 24 hours and then stained with PI. Data present the mean ± SD for altered percentages of subG1 populations in different breast cancer cells treated with 10 μg/ml of butein for 24 hours **(D)**. Cell populations at different stages of cell cycle were presented as 100% rate **(E)**. Experiments were performed in triplicate.

Next, we examined butein effect on ROS generation in different breast cancer cell lines. Butein at 10 μg/ml for 5 minutes reduced ROS levels in almost all breast cancer cell lines, whereas it did not affect those in luminal HER2^+^ HCC-1419, HCC-2218 and SKBR-3 cells (Figure 3A and B). Interestingly, basal ROS levels in butein-resistant and luminal HER2^+^ HCC-1419, HCC-2218 and SKBR-3 breast cancer cells were lower than those in butein-sensitive breast cancer cells including HER2^+^ BT-474 (luminal, ER^+^/PR^+^/HER2^+^) and HCC-1569 (basal A, HER2^+^) cells (Figure 3A). Overall, butein effect on the alteration of ROS level might be one of key steps for butein-induced apoptosis of breast cancer cells, which was confirmed by a correlation between its reduction of ROS levels and induction of apoptotic cell deaths (Figure 3C). Next, we examined butein effect on cell cycle. Butein did not commonly affect cell cycle profiles, when cells were treated with 10 μg/ml of butein for 24 hours. Consistently, butein caused the increase of subG1 population in different breast cancer cells, whereas it did not accumulate luminal HER2^+^ HCC-1419, HCC-2218 and SKBR-3 breast cancer cells at subG1 phase (Figure 3D). However, butein did not commonly affect cell cycle in different breast cancer cells (Figure 3E). Therefore, butein effect on apoptosis appears to be related with the alteration of ROS level.

### Butein inhibits AKT phosphorylation

**Figure 4:**
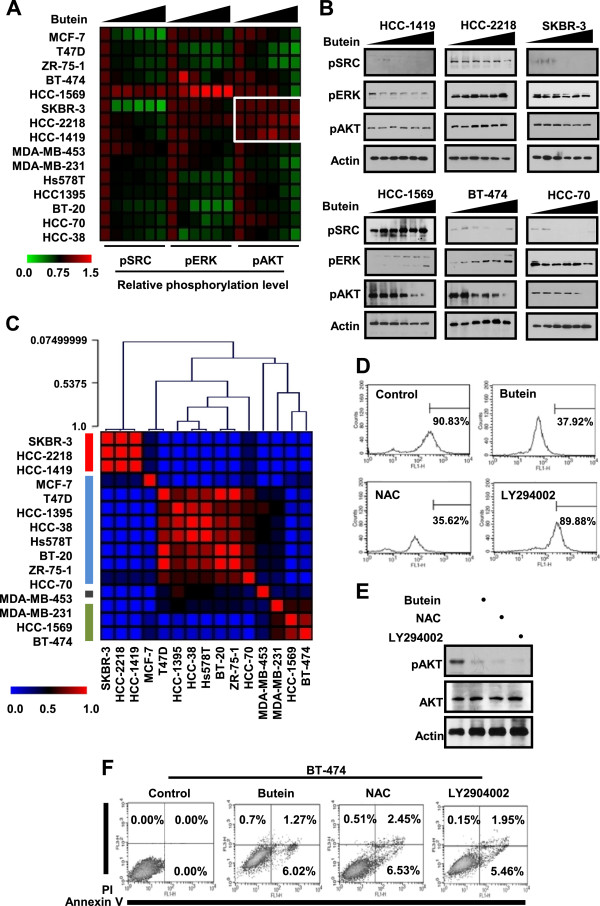
**Butein effect on AKT phosphorylation. (A)** Cells were treated with 10 μg/ml of butein for 15 minutes. Relative phosphorylation levels of SRC, ERK and AKT in different breast cancer cell lines were presented in heatmap. **(B)** Representative data for phosphorylation levels of SRC, ERK, and AKT. Actin was detected as a loading control. **(C)** NMF analysis to categorize breast cancer cells based on butein-altered phosphorylation levels of SRC, ERK and AKT. (D-E) Cells were treated with 40 μg/ml of butein, 40 mM of NAC or 10 μM of LY294002 for 5 minutes. ROS levels were measured by incubating cells with H_2_DCF-DA for another 1 hour **(D)**, and AKT phosphorylation was detected by anti-p-AKT antibody **(E)**. **(F)** Cells were treated with NAC at 40 mM, LY294002 at 10 μM or butein at 10 μg/ml for 24 hours and then stained with Annexin V and PI. Apoptotic cells were measured by flow cytometry.

Next, we examined butein effects on SRC, ERK and AKT. When cells were treated with different doses of butein for 15 minutes, butein did not uniquely affect phosphorylation of either SRC or ERK in different breast cancer cells (Figure 4A), while butein inhibition of SRC has been revealed [1]. However, butein inhibition of AKT phosphorylation was uniquely detected in various breast cancer cells including butein-sensitive HCC-1569 (basal A, HER2^+^), BT-474 (luminal, ER^+^/PR^+^/HER2^+^), and HCC-70 (basal A, TNBC) cells, while it was not found in butein-resistant and luminal HER2^+^ SKBR-3, HCC-1419 and HCC-2218 cells (Figure 4A and B). When we investigated patterns of cell lines based on butein-altered phosphorylation levels of SRC, ERK and AKT using NMF analysis, four different clusters were hierarchically categorized (Figure 4C). Interestingly, butein-resistant HER2^+^ SKBR-3, HCC-1419 and HCC-2218 cell lines were clustered in the same group, which is similar to results from butein effect on ROS reduction and apoptotic cell death. Thus, our data demonstrate that butein inhibition of AKT phosphorylation is related with apoptosis of different breast cancer cells except for butein-resistant and luminal HER2^+^ HCC-1419, HCC-2218 and SKBR-3 cells.

Next, we examined that butein inhibition of AKT phosphorylation was related to its reduction of ROS levels. When butein-sensitive BT-474 cells were treated with butein at 10 μg/ml, N-acetyl-L-cystein (NAC) at 40 mM or AKT inhibitor, LY294002 at 10 μM for 5 minutes, LY294002 failed to reduce ROS level, whereas butein and NAC reduced both ROS level (Figure 4D) and AKT phosphorylation (Figure 4E). Thus, our data indicate that butein reduction of ROS levels leads to the decrease of AKT phosphorylation. Furthermore, both NAC at 40 mM and LY294002 at 10 μM caused apoptosis of HER2^+^ BT-474 cells (Figure 4F). To confirm that butein reduction of ROS production affected cell viability by inhibiting AKT phosphorylation, butein-sensitive and -resistant breast cancer cells were treated with NAC or homobutein (a structural similarity between homobutein and butein = 0.914) [40]. Whereas NAC effect on cell viability was similar to butein, homobutein did not affect cell viability and ROS production (Additional file 1: Figure S1 and S2). Moreover, HER2 silencing did not alter butein sensitivity in butein-resistant HER2^+^ luminal HCC-1419 and SKBR-3 cells (Additional file 1: Figure S3), suggesting that HER2 expression does not determine butein sensitivity in HER2^+^ breast cancer subtype. Therefore, our data indicate that butein inhibitions of ROS generation and AKT phosphorylation are crucial for apoptotic cell death.

### Butein inhibits ROS production and AKT phosphorylation in vivo

**Figure 5:**
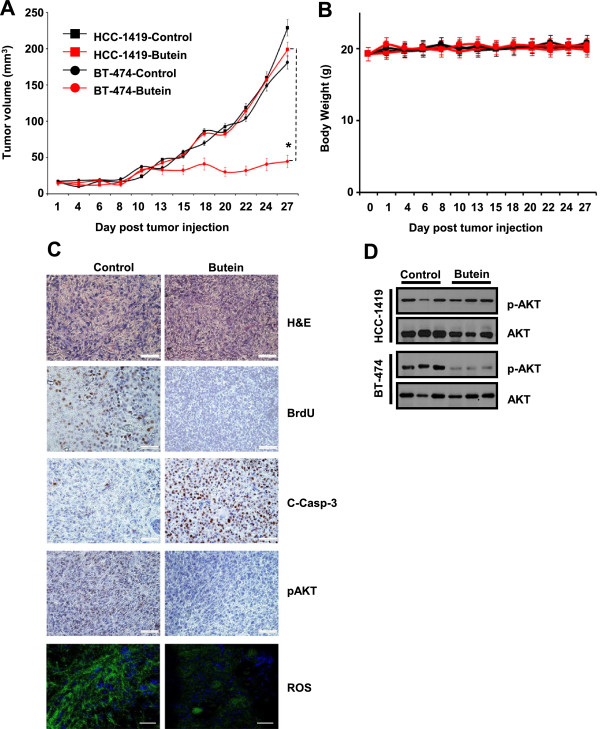
**Butein inhibition of both ROS production and AKT phosphorylation *in vivo*. (A)** Tumor cells were orthotopically injected into nude mice (n = 5/group). Butein (10 μg/ml) or saline was subcutaneously injected six days after orthotopic tumor injection, which was repeated three times a week. Tumor volumes were measured at indicative time points. Asterisks indicate that P-value is less than 0.05. **(B)** Mouse body weights were measured at indicative time points. **(C)** Histopathological observations. BrdU, cleaved Caspase-3 and pAKT were stained with appropriate antibodies. *In situ* ROS was labeled with H_2_DCF-DA. A bar indicates a scale of 40X observation. **(D)** Levels of phosphorylated and total AKT in xenograft tumor tissues.

Next, we examined whether butein inhibited breast cancer growth *in vivo*. Butein-sensitive HER2^+^ BT-474 or butein-resistant HER2^+^ HCC-1419 cells were injected into the 4^th^ left mammary fat pad of nude mice, and then 10 μg/ml of butein was subcutaneously injected two times a week. Butein inhibited orthotopic BT-474 tumor growth, whereas it failed to inhibit orthotopic HCC-1419 tumor growth (Figure 5A). In addition, it did not affect body weights (Figure 5B). Next, we examined if butein affects tumor cell proliferation and apoptosis *in vivo*. BrdU-incorporation assays showed that butein reduced BrdU-positive tumor cell numbers in BT-474 tumor cohorts (Figure 5C). Furthermore, butein increased cleaved caspase-3-positive tumor cell numbers in BT-474 tumor cohorts (Figure 5C). Therefore, our data indicate that butein inhibits *in vivo* breast tumor growth by causing apoptosis.

**Figure 6:**
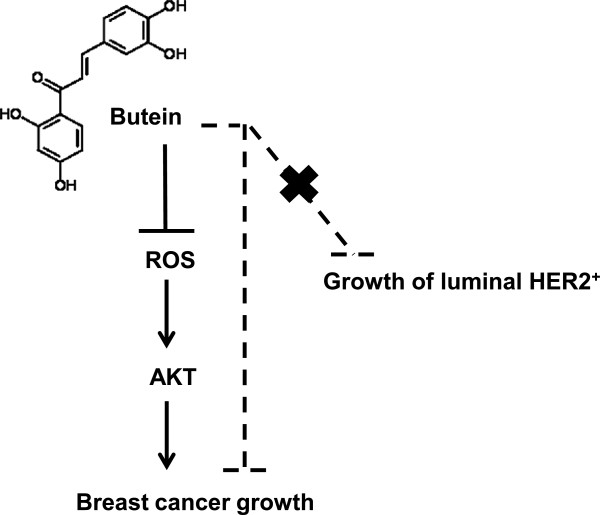
**A cartoon to summarize butein effect on breast cancer cells.** Butein reduces intracellular ROS level, which inhibits AKT phosphorylation. Butein inhibition of ROS production and AKT phosphorylation results in the suppression of breast cancer growth. While breast cancer cells tested here are sensitive to butein, luminal HER2^+^ SKBR-3 and HCC-1419 and HCC-2218 cells are resistant to butein. This resistant phenotype may be related with basal ROS level.

To confirm butein effect on ROS generation and AKT phosphorylation *in vivo*, levels of ROS and phosphorylated AKT were examined in xenograft tumor tissues. Butein reduced ROS-producing tumor cell numbers *in situ*, when DCF-fluorescent tumor cells were counted in butein-sensitive BT-474 tumor tissues treated with either saline or butein (Figure 5C). Therefore, butein decreases ROS level *in vivo*. Likewise, butein reduced the level of phosphorylated AKT in butein-sensitive HER2^+^ BT-474 cells but not in butein-resistant HER2^+^ HCC-1419 cells (Figure 5C and D). Thus, our *in vivo* data confirmed anti-cancer effect of butein via reductions of ROS level and AKT phosphorylation (Figure 6).

## Discussion

Butein has long been studied in various disease model systems including cancer [3-7,9,11,13,14,18,20-22,24]. Recent researches have revealed that butein inhibited breast cancer cell proliferation, migration and invasion in different types of breast cancer cells [8,13,14]. Those previous findings suggested that butein might have diverse roles in different types of breast cancer cells. However, it was still unclear whether butein effect was limited in particular breast cancer cell lines previously examined, or could be generalized even in certain types of breast cancer cells. So, we examined butein effects on various breast cancer cells, and found that common effects of butein against different breast cancer cells.

Our data show that butein reduction of ROS level is a key step to induce the apoptosis in various breast cancer cells. Consistently, NAC caused apoptosis of butein-sensitive breast cancer cells, which is in line with a previous report that NAC prevents leukemia [41]. Thus, ROS homeostasis is likely to be crucial for maintaining the viability even in cancer cells. Interestingly, we found that butein effect on alterations of ROS levels is likely to be dependent on environmental cues. In detail, when cells were treated with butein in serum-depleted medium, ROS level was slightly and transiently increased (data not shown). Therefore, butein effect on ROS levels may be dependent on environmental cues such as certain factors contained in serum, while we could not find more clear procedures from previous reports that showed a dramatic induction of ROS generation by butein [14,31]. Those findings suggest that butein may play pleiotropic roles in tumor microenvironment-dependent manner. Meanwhile, when MCF-7 cells were exposed to chronic oxidative stress such as H_2_O_2_, the increased ROS level led to their tumorigenic potentials *in vitro*[42]. Those data indicate that ROS might have both positive and negative roles, while it has been known to cause apoptosis. Therefore, as we found herein, butein inhibition of ROS level is likely to suppress cancer development.

While butein has been revealed to affect SRC via direct interactions [1], we could not find common inhibitory effects of butein on SRC or its downstream ERK in various breast cancer cell lines. In addition, we failed to find any common effect of butein on activities of RhoGTPases (data not shown). Our data rather suggest that butein effect on breast cancer cells is dependent on its inhibition of AKT phosphorylation, while it was not found in luminal HER2^+^ breast cancer cell lines such as SKBR-3, HCC-1419 and HCC-2218 cells. Our NMF analysis based on phosphorylation status of SRC, ERK and AKT also showed that butein-resistant luminal HER2^+^ breast cancer cell lines were categorized in the same hierarchical cluster. Therefore, butein inhibition of AKT would be one of readouts for butein sensitivity. However, our preliminary studies indicate that butein inhibition of AKT phosphorylation may be not unique in other cancer cell lines such as prostate cancer cell lines and leukemic cell lines (unpublished data). Therefore, butein inhibition of AKT phosphorylation is likely to be restricted to breast cancer cells except for luminal HER2^+^ breast cancer cells. Moreover, butein-mediated imbalance of ROS levels resulted in the inhibition of AKT phosphorylation. Thus, it is plausible that the cytotoxic effect of butein may be evoked by inhibiting ROS production and AKT phosphorylation. Consistently, we found that butein suppression of breast cancer growth was correlated with its reduction of levels of both ROS and phosphorylated AKT, *in vivo*. Meanwhile, butein inhibition of AKT phosphorylation was not recovered until 24 hours after butein treatment (data not shown), which indicate that butein inhibition of AKT phosphorylation may be irreversible. Recent studies have revealed ROS regulation of AKT, *vice versa*[43,44]. Therefore, it is plausible that butein may block a signaling circuit involving ROS and AKT to decide cell fate. Meanwhile, homobutein failed to show similar effects of butein on breast cancer cells, while they are chalcone derivatives in highly similar structures and target the same molecules, HDAC and NF-κB [40]. Therefore, butein specifically targets ROS and AKT in breast cancer cell death, while molecular mechanisms by which butein regulates ROS are still intriguing.

Meanwhile, basal ROS levels were lower in butein-resistant luminal HER2^+^ SKBR-3, HCC-1419 and HCC-2218 breast cancer cells than in other cell lines including HER2^+^ BT-474 and HCC-1569 cells, while basal ROS levels in different breast cancer cells appeared to be independent of breast cancer subtypes. Therefore, it is possible that basal ROS levels may intrinsically determine butein sensitivity. Accordingly, it is plausible that alterations of tumor microenvironment may affect ROS levels in cancer cells and further regulate tumor growth and metastasis [45,46]. This notion is in line with our assumption about different butein sensitivities of HER2^+^ breast cancer cells. As ROS and AKT are involved in breast cancer metastasis [37,47,48], environmental forces for tumor development and metastasis may modulate ROS and AKT in cancer cells, *vice versa*. Meanwhile, ROS appears to force endocrine therapy resistance as well as tumorigenicity in ER^+^ breast cancer, as glucose oxidase-mediated ROS induction led to the phosphorylation and downregulation of ERα in MCF-7 [42,49]. Likewise, ROS appears to regulate both the growth and invasiveness of TNBC cells [50,51]. ROS regulated both the migration and invasion of HER2^+^ SKBR-3 cells [37], which is in line with the finding that butein inhibited SKBR-3 cell migration and invasion [8]. Thus, while our study revealed that butein failed to cause apoptosis of luminal HER2^+^ breast cancer cells, it might block metastatic abilities of those cell types. However, it is still unclear why luminal HER2^+^ breast cancer cells are resistant to butein and have relatively low basal ROS levels. In our study, butein-sensitive BT-474 cells are luminal triple-positive (HER2^+^, ER^+^ and PR^+^) and HCC-1569 cells are basal A HER2^+^. Therefore, different subcategories of HER2^+^ breast cancer cells may exhibit different butein sensitivity, while we still do not know determinants for those subcategories. In our study, HER2 itself did not affect butein sensitivity, as HER2 silencing did not alter butein sensitivity in butein-resistant HER2^+^ breast cancer cells. Regarding to that issue, our ongoing study will explore to address intrinsic factors causing different butein sensitivities associated with ROS productions in HER2^+^ breast cancer cells.

## Conclusions

In conclusion, our study demonstrates that butein inhibits breast cancer growth through reducing ROS level and AKT phosphorylation. Therefore, our finding provides better knowledge for butein effect on breast cancer and also suggests its treatment option. Meanwhile, we failed to find a synergistic effect of butein with one of chemotherapeutics, doxorubicin (data not shown). However, as herbal medicines are widely used to prevent or to treat diseases including cancer, it is worth finding a synergism between butein and anti-cancer drugs already known. Synergistic effects are expected to reduce adverse effects of anti-cancer drugs. In addition, our study suggests that ROS level is likely to determine characteristics of HER2^+^ breast cancer cells, while these are yet defined clearly. Therefore, this finding is also expected to improve our knowledge for ROS biology especially in breast cancer.

## Competing interests

The authors declare that they have no competing interests.

## Authors’ contributions

CSG designed the study, performed experiments and wrote the manuscript. WSM carried out experiments. KSG participated in the design of the study and supervised researches. All authors read and approved the final manuscript.

## Supplementary Material

Additional file 1 Butein specifically regulates ROS production in breast cancer cells. Click here for file
