# Supplementary material for: Butein suppresses breast cancer growth by reducing a production of intracellular reactive oxygen species
Source: J Exp Clin Cancer Res. 2014 Jun 11;33(1):51. doi: 10.1186/1756-9966-33-51 (PMC4064524; doi:10.1186/1756-9966-33-51)
Supplement: Additional file 1 — Butein specifically regulates ROS production in breast cancer cells. [file 1756-9966-33-51-S1.pdf]

Supplementary data

Butein suppresses breast cancer growth by reducing a production of intracellular reactive oxygen species

Sung-Gook Cho, Sang-Mi Woo, Seong-Gyu Ko[§]

Department of Preventive Medicine, College of Korean Medicine, Kyung Hee University, 1 Hoegi, Seoul, 130701, Korea

[§]Corresponding author: Seong-Gyu Ko

Email addresses: Cho SG: chosg@khu.ac.kr, Woo SM: taoya@hanmail.net, Ko SG: epiko@khu.ac.kr

Supplementary materials and methods

Cell viability assays For cell viability, cells were cultured in 96 well plates, treated with different concentrations of homobutein or NAC, and subjected to the MTT assays. Experiments were performed in quadruplicate and repeated three times independently. Data were presented as the mean ± SEM. P-values less than 0.05 were considered statistically significant. Homobutein was obtained from Extrasynthese (Genay Cedex, France).

ROS detection Cells were treated with homobutein for 5 minutes and then treated with H_2DCF-DA for another 1 hour. Flow cytometry experiments were performed in triplicate and repeated three times independently.

HER2 silencing HER2 expression was silenced in the cell by transfecting HER2 siRNA for 48 hours. Control and HER2 siRNAs were obtained from Santa Cruz Biotechnology (CA, USA). Western blot for HER2 determined its silencing.

Supplementary figure legends

Figure S1. Homobutein did not affect intracellular ROS level and cell viability. (A) Homobutein structure. (B) Cells were treated with different concentrations of homobutein (1ng/ml to 100μg/ml) for 48 hours and then subjected to the MTT assays. (C) Cells were treated with homobutein at 10μg/ml for 5 minutes and incubated with H_2DCF-DA for another 1 hour. ROS levels were measured using flow cytometry. Experiments were independently repeated three times.

Figure S2. NAC reduces the viability of breast cancer cells. Cells were treated with different concentrations of NAC for 48 hours and then subjected to the MTT assays.

Figure S3. HER2 does not affect butein sensitivity in butein-resistant HER2+ breast cancer cells. SKBR-3 or HCC-1419 cells were transfected with control or HER2 siRNAs for 24 hours (A), and then treated with butein for another 48 hours to examine cell viabilities (B).

Figure S1

A

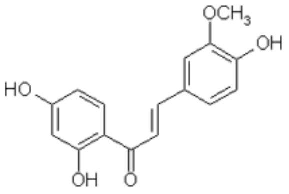

B

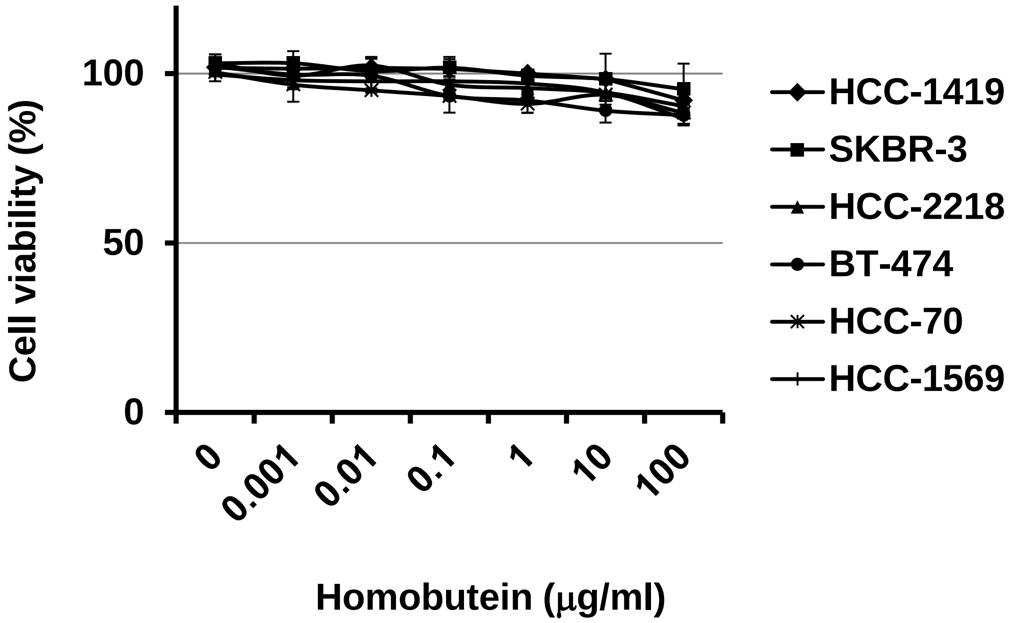

C

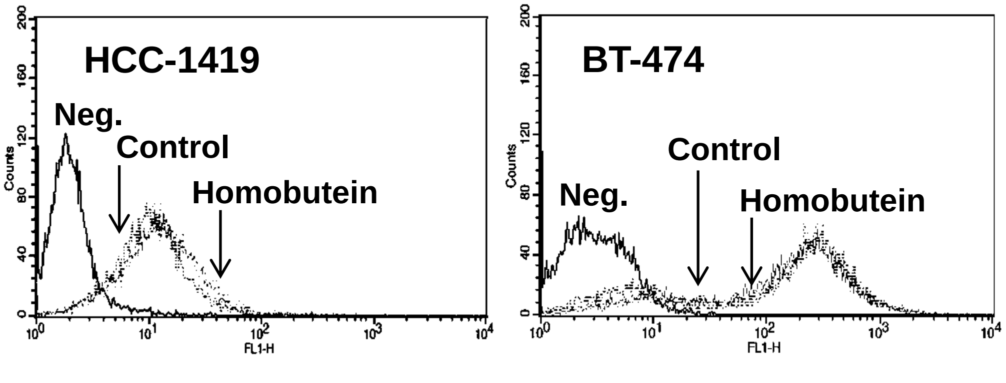

Figure S2

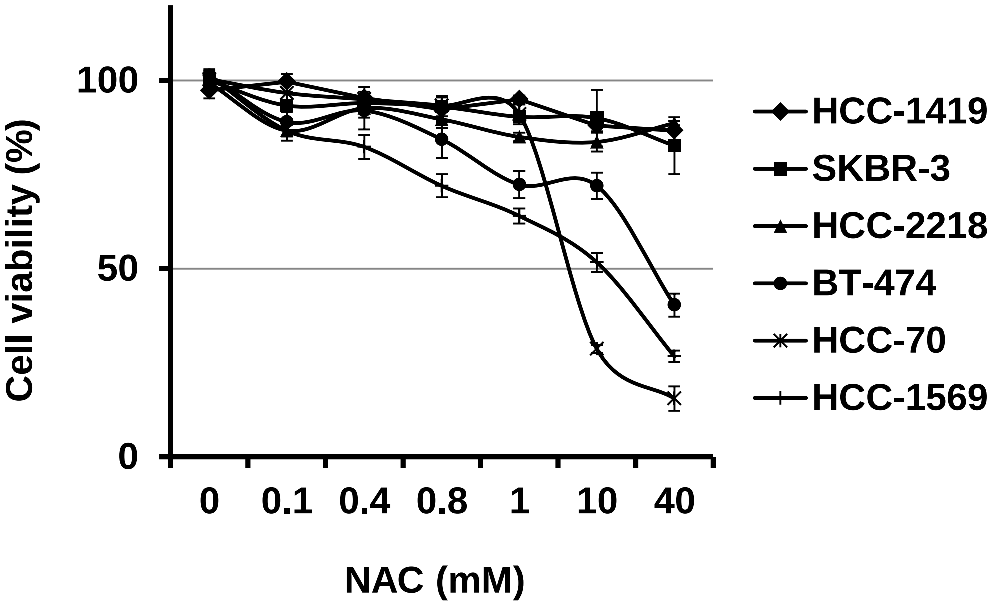

Cell viability (%)
100
50
0
0
0.1
0.4
0.8
1
10
40
NAC (mM)
HCC-1419
SKBR-3
HCC-2218
BT-474
HCC-70
HCC-1569

Figure S3

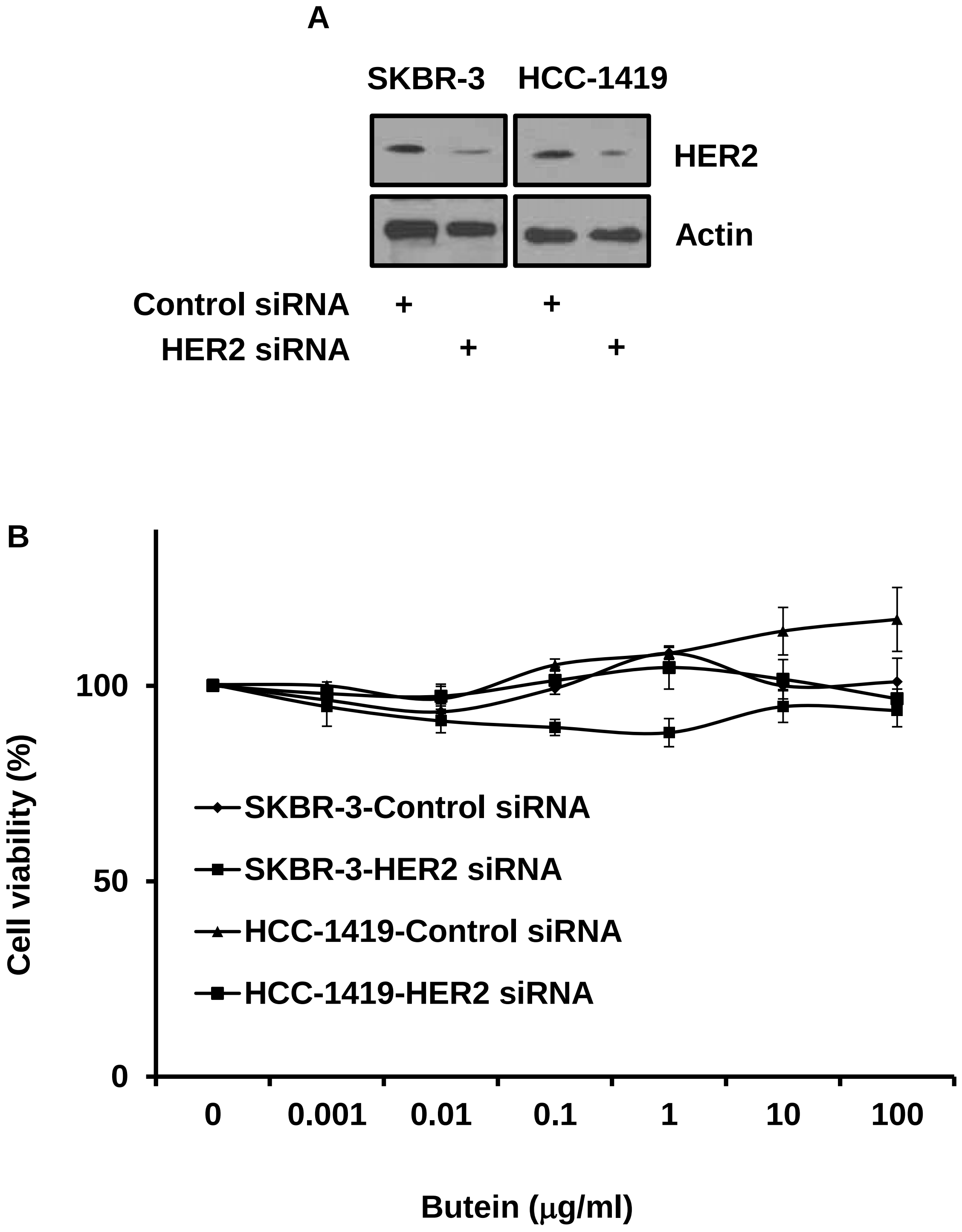
A
SKBR-3
HCC-1419
HER2
Actin
Control siRNA
HER2 siRNA
+
+
+
+
B
Cell viability (%)
100
50
0
SKBR-3-Control siRNA
SKBR-3-HER2 siRNA
HCC-1419-Control siRNA
HCC-1419-HER2 siRNA
0
0.001
0.01
0.1
1
10
100
Butein (μg/ml)
